# Cardiac Cell Membrane-Coated Nanoparticles as a Potential Targeted Delivery System for Cardiac Therapy

**DOI:** 10.3390/biomimetics10030141

**Published:** 2025-02-25

**Authors:** Faprathan Pikwong, Jiraporn Kamsarn, Wattanased Jarisarapurin, Phornsawat Baipaywad, Hansoo Park, Sarawut Kumphune

**Affiliations:** 1 Biomedical Engineering Institute, Chiang Mai University, Chiang Mai 50200, Thailand; faprathan_p@cmu.ac.th (F.P.); jiraporn_kam@cmu.ac.th (J.K.); wattanased.j@cmu.ac.th (W.J.); phornsawat.b@cmu.ac.th (P.B.); 2 Biomedical Engineering and Innovation Research Centre, Chiang Mai University, Mueang Chiang Mai District, Chiang Mai 50200, Thailand; 3 Office of Research Administration, Chiang Mai University, Chiang Mai 50200, Thailand; 4 School of Integrative Engineering, Chung-Ang University, Seoul 06974, Republic of Korea; heyshoo@cau.ac.kr

**Keywords:** targeted drug delivery, cardiovascular diseases, nanoparticles, cardiac cells, cell membrane-coated nanoparticles, mesoporous silica nanoparticles

## Abstract

Cardiomyopathies, a cause of heart failure, are a predominant cause of death globally and may lead to discernible myocardial abnormalities. Several therapeutic agents were discovered, developed, investigated, and evaluated to save patients’ lives and improve their quality of life. The effective administration of drugs improves therapeutic outcomes while reducing side effects. Nanoparticles (NPs) have been utilised for the delivery of therapeutic agents and demonstrate promise in reducing myocardial ischaemia/reperfusion injury. However, significant limitations of NPs include non-specific targeting and immunogenicity. To improve cardiac targeting and biocompatibility, surface modifications using a cardiac cell membrane (cCM) coating on the surface of NPs have been hypothesised. Here, cCMs were isolated from the human ventricular cell line (AC16), and mesoporous silica nanoparticles (MSNs) were synthesised and then coated with cCMs. The cardiac cell membrane-coated mesoporous silica nanoparticles (cCMCMSNs) did not significantly alter the encapsulation efficiency or the release profile of the loaded drug (Rhodamine B) in comparison to MSN. Moreover, cCMCMSNs demonstrated a significantly enhanced distribution of RhB specifically to cardiac cells, compared to other cell types, without causing cytotoxicity. To evaluate immune escape, cCMCMSNs were exposed to activated macrophages, demonstrating that cCMCMSNs were phagocytosed to a lesser extent than MSN. This study demonstrated the synthesis of cardiac cell membranes coated on the surface of nanoparticles as nanomedicine technologies that enhance selective drug delivery to cardiac cells, potentially offering an alternate method for drug administration in cardiovascular diseases.

## 1. Introduction

Cardiomyopathies are a group of heart muscle disorders that frequently cause heart failure (HF), a major cause of mortality and morbidity, accounting for an important amount of global health care expenses [1,2]. Cardiomyopathy is classified into two categories: primary, attributable to hereditary causes, and secondary, resulting from inflammation and expressing as dilated, hypertrophic, or restricted patterns [3]. The American Heart Association (AHA) defines it as a heterogeneous group of myocardial disorders, usually characterised by inappropriate ventricular hypertrophy or dilatation [4]. One of the most severe cardiomyopathies is ischaemic cardiomyopathy (ICM), which represents the heart’s impaired capacity to effectively pump blood, resulting from myocardial injury caused by ischaemia [5]. Innovative therapeutic strategies that can successfully address the underlying causes and improve cardiac repair are becoming increasingly necessary as the understanding of cardiomyopathy advances. Cardiac treatment, involving the administration of drugs, growth factors, or bioactive substances to damage myocardial tissue, has shown significant potential [6]. The lack of effective and secure delivery methods exhibits a challenge to the clinical use of cardiac medication.

Nanomaterials have shown great potential in a variety of fields, including the reduction of off-target adverse effects, the enhancement of diagnostic and therapeutic efficacy, and the reduction of long-term toxicity [7]. Nanoparticles (NPs) have been used for drug delivery into the bloodstream [8]. NPs have essential limitations when it comes to targeting certain cells or organs, especially cardiac tissue [9]. There is a possibility that a surface modification of NPs could increase their selectivity for particular cell types [10].

**Scheme 1:**
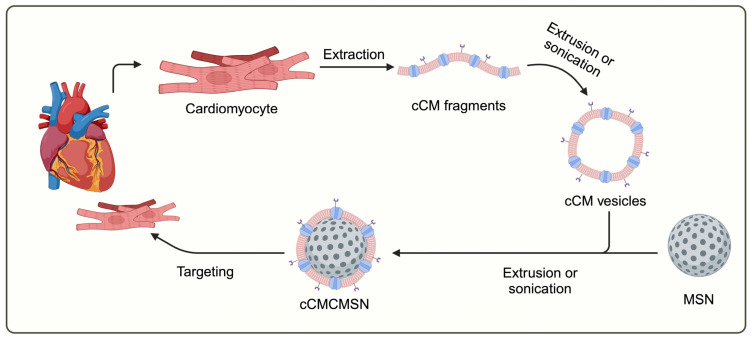
Schematic of preparation of cardiac cell membrane-coated mesoporous porous silica nanoparticles (cCMCMSNs).

The surface modification by coating NPs with layers of cell membranes (CMs) has been reported [8,11]. Cell membrane-coated NPs (CMCNPs) are a novel type of biomimetic NPs that incorporate cell membranes with engineered NPs for effective therapeutic drug delivery [12]. CMCNPs have been studied in cardiovascular disease (CVD) with cell membranes from various cell types, including red blood cells (RBCs), immune cells (WBCs), platelets, cancer cells, and stem cells [13,14,15,16,17,18]. In a previous study, the therapeutic potential of CMCNPs was demonstrated using mesenchymal stem cell (MSC) membrane-coated mesoporous silica NPs (MSN) to deliver the microRNA-21 (miR-21), reduce the infarct size, and improve cardiac function in mice subjected to a myocardial infarction model [18]. Although the application of MSCs membrane-coated NPs might offer cardioprotection, their lack of specificity for cardiac tissue is apparent. This lack of specificity comes from a non-specificity of stem cells for cardiac cells. Therefore, the utilisation of cardiac cell membrane (cCM)-coated MSNs (cCMCMSNs) (Scheme 1) could provide the specificity for drug delivery to cardiac cells given that cCMs possess unique surface proteins known as homing proteins, which can selectively interact with cardiac cell receptors. These properties could enable specific delivery of therapeutic agents to the heart tissue.

## 2. Materials and Methods

### 2.1. Chemicals and Reagents

Tetraethyl orthosilicate (TEOS, 98.0%), cetyltrimethyl ammonium bromide (CTAB), Tris-HCl, magnesium sulphate, and ethanol were purchased from Sigma-Aldrich (Milwaukee, WI, USA). Sodium hydroxide and hydrochloric acid were purchased from RCI Labscan (RCI Labscan Ltd., Bangkok, Thailand). Dulbecco’s modified Eagle medium (DMEM), foetal bovine serum (FBS), penicillin, streptomycin, and trypsin-EDTA were purchased from Gibco (Gibco BRL; Life Technologies, Inc., New York, NY, USA).

### 2.2. Cell and Cell Culture

Adult human ventricular myocyte cell line cells (AC16, ATCC-CRL3568), adenocarcinoma human alveolar basal epithelial cells (A549, CCL-185), human hepatoma cell lines (Hep G2, HB-8065), and a mouse macrophagic cell line (RAW 264.7, TIB-71) were purchased from American Type Cell Culture. Cells were cultivated in the suggested culture medium from ATCC, supplemented with 10% (*v*/*v*) foetal bovine serum (FBS) and 100 units/mL of penicillin/streptomycin. Cells were cultured at 37 °C in a humidified atmosphere of 95% air and 5% carbon dioxide.

### 2.3. Preparation of Mesoporous Silica Nanoparticles (MSNs)

To prepare NPs, MSNs were synthesised using a modified Stöber method [19]. First, CTAB (100 mg) was added to 50 mL of DI water and 1 mL of ethanol while stirring at 750 rpm at 50 °C for 20 min. After that, TEOS (575 µL) and NaOH (350 µL, 2 M) were added to the solution, stirring at 750 rpm at 50 °C for 2 h. Then, the solution was incubated at room temperature until reaching 25 °C. After the incubation, the solution was centrifuged at 8000 rpm for 10 min. Then, the pellet was extracted twice using a solution of HCl and ethanol, followed by three centrifugations at 8000 rpm for 10 min. After centrifugation, the pellet was washed three times in a solution with an ethanol: DI water ratio of 50:50, 80:20, and 100:0 at 8000 rpm for 10 min. Finally, the pellet was resuspended with DI water and dried with a freeze-drying process.

### 2.4. Preparation of cCMCMSNs

The Scheme 1 shows the conceptualized information on preparation of cCMCMSNs. The extraction technique was modified from Jang Y et al. [20]. The cardiac cell line (AC16) was cultured in a T-175 flask. At 80% confluence, cells were harvested using trypsin-EDTA. The cells were resuspended with 10 mL of TM buffer (50 mM Tris-HCL, pH 7.4, 10 mM magnesium sulphate, and a tablet of EDTA-free protease inhibitor). Cells were homogenised using a probe homogeniser at 6000 rpm for 5 min, followed by sonication at 150 W for 2 s, repeated for 5 min. Then, the solution was centrifuged at 100,000× *g* at 4 °C for 1 h. The supernatant was discarded, and the pellet was collected. The cell membranes were measured using a Bradford assay. For coating, MSNs were incubated with the cell membranes in a mass ratio of 2:1. The solution was sonicated by the probe sonicator at 150 W plus 2 s for 5 min. After that, the solution was centrifuged at 10,000× *g* for 10 min at 4 °C. The supernatant was removed, and the pellet was resuspended with a PBS solution.

### 2.5. Physical Characteristics of Nanoparticles

The NPs were characterised by size, polydispersity index (DPI), and zeta potential using a Zetasizer (Malvern, England). The porousness and surface area of the MSNs were calculated using the Brunauer–Emmett–Teller (BET) theory. NP morphology was determined using the transmission electron microscope (TEM) JEM 2010 at 100 kV; the NPs were fixed with 2% glutaraldehyde for 30 min and dropped onto an EMS CF400-Cu-50 grid. After that, the NPs were stained with an Uranyless solution for 1 min, followed by TEM examination.

### 2.6. Determination of Cell Membrane Proteins Using Sodium Dodecyl Sulphate-Polyacrylamide Gel Electrophoresis (SDS-PAGE)

The lysate cells, MSNs, and cCMCMSNs were treated with 2× SDS sample buffer and boiled at 95 °C for 5 min. After that, samples were subject to 10% polyacrylamide gel electrophoresis. After electrophoresis, the gel was incubated with a gel-fixing buffer that contained 50% (*v*/*v*) of ethanol and 10% (*v*/*v*) of acetic acid for 1 h. After incubation, the gel was washed with a washing buffer that contained 50% (*v*/*v*) of methanol and 10% (*v*/*v*) of acetic acid overnight. The gel was stained with Coomassie Brilliant Blue R250 for 4 h and appropriately destained until clear.

### 2.7. Encapsulation and Release of Nanoparticles

To load fluorescence into NPs, MSNs (10 mg) were treated with rhodamine B (0.04 mM) as a fluorescent agent for 24 h at room temperature. After incubation, cCMs were added to the solution, which was subsequently sonicated at 150 W for 2 s every 5 min. The NPs were rinsed twice with DI water before being centrifuged at 10,000 rpm for 10 min three times. To test the effectiveness of encapsulation, the supernatant was collected, and rhodamine B was detected at 554 nm using an UV–Vis spectrophotometer. NPs were incubated with PBS for 30 min to 72 h to assess their release patterns. After incubation, the solution was centrifuged, yielding 500 µL of supernatant. The solution was then diluted with 500 µL of PBS. The UV–Vis spectrophotometer set to 554 nm was used to determine the quantity of rhodamine B in the collected supernatant.

### 2.8. Human Cardiomyocyte Cytotoxicity

The cytotoxicity was determined using a MTT viability assay. The AC16 cells were seeded on a 96-well plate at a density of 10,000 cells per well and incubated for 24 h. The DMEM was removed, and samples of NPs were added at concentrations of 1, 10, and 100 µg/mL, including MSNs, cCMs, and cCMCMSNs. The experiment was incubated for 24 h at 37 °C. After incubation, the DMEM was removed, and MTT solution (0.5 mg/mL) was added for 2 h at 37 °C. After that, the MTT solution was removed, and DMSO was added to extract formazan. The cell viability was measured using a TECAN infinite m^®^plex microplate reader (Männedorf, Switzerland).

### 2.9. Cell Membrane Staining

PKH26 dye (Sigma-Aldrich, Milwaukee, WI, USA) was employed to stain cCMCMSNs. One microliter of PKH26 dyes was added to 250 μL of diluent C. Subsequently, the diluted PKH26 was mixed with the diluted cCMCMSNs and incubated for 5 min. After incubation, filtered FBS was used to stop staining. The solution was centrifuged at 10,000 rpm for 10 min. The PKH26-cCMCMSN pellet was resuspended in PBS and centrifuged at 10,000 rpm for 10 min. Finally, the PKH26-cCMCMSN pellet was resuspended in DMEM before adding to AC16 cells.

### 2.10. Determination of Cellular Uptake

The AC16 cells were seeded on a 96-well plate at a density of 10,000 cells per well and incubated for 24 h. The DMEM was removed, and then RhB-cCMCMSNs and PKH26-cCMCMSNs were added at 25 µg/mL and incubated for 4 h. After incubation, the DMEM was removed and rinsed with PBS twice. The cell was fixed using a 4% paraformaldehyde solution for 30 min. The cells were stained with DAPI for 20 min and rinsed with PBS twice. The internalisation was detected using an EVOS M5000 fluorescence microscope (Thermo Fisher Scientific Inc., Waltham, MA, USA) to record fluorescent pictures of the cells. A quantification of internalisation was evaluated by counting cells as a percentage of cellular uptake.

### 2.11. An In Vitro Selective Delivery of Nanoparticles

The A549 and Hep G2 cells were seeded onto a 96-well plate at a density of 10,000 cells per well and incubated for 24 h. The DMEM was removed, then RhB-cCMCMSNs were added at 25 µg/mL and incubated for 4 h. After incubation, the DMEM was removed and rinsed with PBS twice. The cells were fixed using a 4% paraformaldehyde solution for 30 min. The cells were stained with DAPI for 20 min and rinsed with PBS twice. The internalisation was detected using an EVOS M5000 fluorescence microscope (Thermo Fisher Scientific Inc., Waltham, MA, USA) to record fluorescent pictures of the cells. The quantification of internalisation was evaluated by counting cells as a percentage of cellular uptake.

### 2.12. Immune Escape

Mouse macrophagic RAW 264.7 cells were seeded into a 96-well plate at a density of 10,000 cells per well and incubated for 24 h. To induce activated macrophages, 100 mL of 1 µg/mL of bacterial lipopolysaccharide (LPS) (Sigma-Aldrich, Milwaukee, WI, USA) was added to activate the macrophages for 24 h. After that, the DMEM was removed and replaced with RhB-cCMCMSNs at a concentration of 25 µg/mL for 4 h. After incubation, the DMEM was removed and rinsed with PBS twice. The cells were fixed using a 4% paraformaldehyde solution for 30 min. The cells were stained with DAPI for 20 min and rinsed under PBS twice. The internalisation was detected by an EVOS M5000 fluorescence microscope (Thermo Fisher Scientific Inc., Waltham, MA, USA) to record fluorescent pictures of the cells. The quantification of internalisation was evaluated by counting cells as a percentage of cellular uptake.

### 2.13. Statistical Analysis

All data were presented as the mean ± standard deviation (SD). The significance of all comparisons was assessed using an unpaired *t*-test or ANOVA, followed by the Tukey–Kramer test. A significance level of 0.05 was employed to determine statistical significance.

## 3. Results

### 3.1. Physical Characteristics

**Figure 1:**
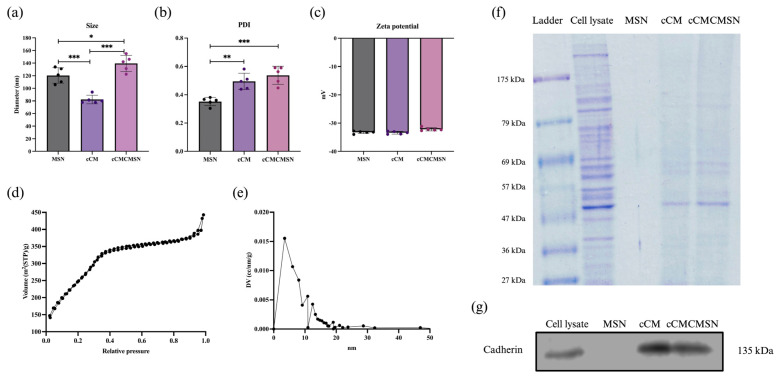
Characteristics of NPs: (**a**) size of NPs, (**b**) PDI of NPs, (**c**) zeta potential of NPs, (**d**) BET diagram of MSN, (**e**) BJH diagram of MSN, (**f**) SDS-PAGE protein separation, and (**g**) the presence of the cardiac cell membrane protein cadherin as shown in a Western blot. * *p* < 0.05, ** *p* < 0.01, *** *p* < 0.001.

The MSNs, cCMs, and cCMCMSNs were assessed to determine their physical characteristics, including size, PDI, and the charge of NPs. Moreover, the pore size of MSNs was measured using BET. cCMCMSNs were confirmed to possess cCM coatings using SDS-PAGE and assessment of pan-cadherin expression using by Western blotting. The results showed that the sizes of MSNs, cCMs, and cCMCMSNs were 120.4 ± 12.4 nm, 82.3 ± 6.8 nm, and 139.4 ± 12.8 nm, respectively (Figure 1a). The polydispersity index (PDI) values were 0.32 ± 0.03, 0.49 ± 0.06, and 0.54 ± 0.06, respectively (Figure 1b). The PDI of MSNs was significantly different compared to that of cCMs and cCMCMSNs. The zeta potential values were −33.26 ± 0.44, −33.40 ± 0.50, and −32.16 ± 0.60, respectively (Figure 1c). The pore size of MSNs was calculated based on the pore-specific surface area using the Brunauer–Emmett–Teller (BET) theory, and the results showed that the value of BET was 18.81 ± 11.25 nm. The results of the nitrogen adsorption-desorption isotherm for MSNs presented a Type IV(a) isotherm according to the IUPAC classification, which indicated a mesopore in the structure (Figure 1d) [21]. The surface area of MSNs was measured at 959.58 m²/g, as indicated by the BET analysis, and the size distribution of the pore is shown in Figure 1e. To determine the successful addition of the cCM coat on MSNs, SDS-PAGE was employed, and the protein content in the sample was visualised using Coomassie staining. The result was shown as the comparison of protein bands between cardiac cell lysate (AC16 cell) and cCMs (Figure 1f). The results showed that the cell lysate, cCMs, and cCMCMSNs exhibited protein bands. However, no proteins were detected in the MSN sample. The protein bands observed in cCMCMSNs showed a similar pattern when compared to those in cCMs. The presence of the cardiac membrane protein cadherin from the incorporation of AC16 cell membranes on cCMCMSNs was identified using Western blot analysis (Figure 1g). The results showed the expression of cadherin protein at a molecular weight of 135 kDa in the cell lysate, cCMs, and cCMCMSNs, while MSNs did not display this band.

### 3.2. Morphology of cCMCMSNs

**Figure 2:**
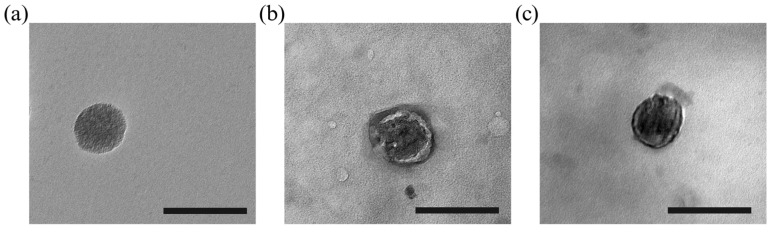
Morphological assessment using TEM: (**a**) MSN, (**b**) cCM, and (**c**) cCMCMSN. Scale bar = 200 nm.

To identify the morphology of NPs, transmission electron microscopy (TEM) was used to analyse MSNs, cCMs, and cCMCMSNs (Figure 2). The results indicated that MSNs had a spherical shape and exhibited porous characteristics (Figure 2a). The cCMs displayed a double-layer membrane with a round shape and hollow particles (Figure 2b). cCMCMSNs also showed a spherical shape, a thick edge, and a smooth surface (Figure 2c). The results suggested that MSNs and cCMCMSNs had similar diameters.

### 3.3. Encapsulation and Release

**Figure 3:**
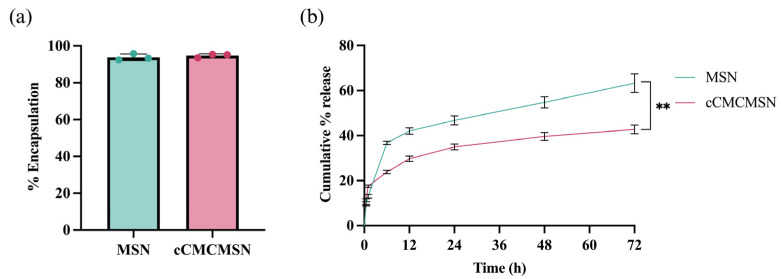
The RhB encapsulation and release profiles of MSNs and cCMCMSNs: (**a**) percentage of encapsulation of RhB, and (**b**) cumulative percentage of RhB release within 72 h. ** *p* < 0.01.

To determine the encapsulation of NPs, RhB was loaded into MSNs using the incubation method [22]. The results showed that the encapsulation efficiencies for MSNs and cCMCMSNs were 93.82 ± 1.78% and 94.71 ± 1.01%, respectively (Figure 3a). There was no significant difference in the encapsulation efficiencies between MSNs and cCMCMSNs. The release profiles of MSNs and cCMCMSNs were collected at endpoints of 30 min and 1, 6, 12, 24, 48, and 72 h. The release percentages were 9.13 ± 0.39%, 13.06 ± 0.80%, 36.77 ± 0.76%, 42.01 ± 1.41%, 46.76 ± 2.02%, 54.75 ± 2.48%, and 63.27 ± 4.16% for MSNs and 11.31 ± 0.69%, 17.53 ± 0.56%, 23.83 ± 0.76%, 29.70 ± 1.19%, 34.98 ± 1.31%, 36.63 ± 1.76%, and 42.75 ± 1.98% for cCMCMSNs, respectively (Figure 3b). The release results at a time point of 72 h showed that cCMCMSNs were released about 20% less than MSNs, representing a significant difference.

### 3.4. In Vitro Cytotoxicity on Cardiomyocytes

**Figure 4:**
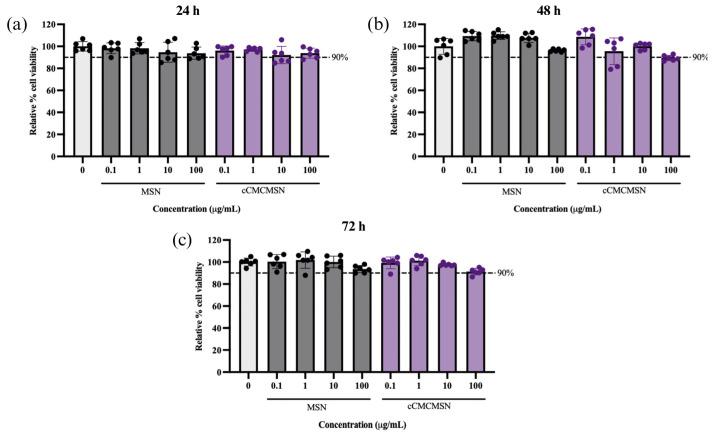
The cytotoxicity of NPs at various concentrations to AC16 cells: (**a**) after a 24-h incubation, (**b**) after a 48-h incubation, and (**c**) after a 72-h incubation.

The cytotoxicity of MSNs and cCMCMSNs was determined using AC16 cells (Figure 4). The result showed that MSNs at concentrations of 0.1, 1, 10, and 100 µg/mL showed 98.26 ± 4.92%, 98.24 ± 5.14%, 94.67 ± 9.10%, and 93.79 ± 5.55% cell viability, respectively, at 24 h. cCMCMSNs at concentrations of 0.1, 1, 10, and 100 µg/mL showed 96.19 ± 4.19%, 97.16 ± 1.75%, 92.11 ± 7.87%, and 93.83 ± 4.56% cell viability, respectively, at 24 h. There were no significant differences in cell viability for both MSNs and cCMCMSNs at every concentration when compared to that of the control group (100.00 ± 4.30%). MSNs showed 109.30 ± 4.24%, 109.80 ± 3.50%, 107.80 ± 4.22%, and 96.28 ± 1.24% cell viability, respectively, at 48 h. cCMCMSNs showed 108.60 ± 7.19%, 95.54 ± 12.10%, 100.10 ± 3.02, and 89.42 ± 2.461% cell viability, respectively, at 48 h. There were no significant differences in cell viability for both MSNs and cCMCMSNs at every concentration when compared to that of the control group (100.00 ± 7.23%). MSNs showed 100.40 ± 6.34%, 101.80 ± 7.43%, 100.10 ± 5.23%, and 93.61 ± 3.24% cell viability, respectively, at 72 h. cCMCMSNs showed 99.26 ± 5.28%, 101.20 ± 4.48%, 97.59 ± 1.20%, and 91.35 ± 2.95% cell viability, respectively, at 72 h. There were no significant differences in cell viability for both MSNs and cCMCMSNs at every concentration when compared to that of the control group (100.00 ± 3.51%). cCMCMSNs exhibited low toxicity to cardiac cells within 72 h of treatment.

### 3.5. Cellular Uptake of cCMCMSNs

**Figure 5:**
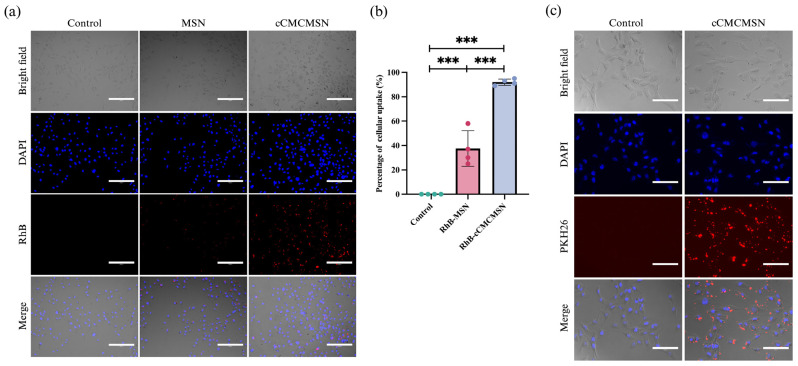
Cellular uptake of RhB-loaded NPs by AC16 cells: (**a**) cellular uptake of MSNs and cCMCMSNs; (**b**) percentage of cellular uptake (scale bar = 300 µm, *** *p* < 0.01); (**c**) cellular uptake of PHK26-stianed cCMCMSNs in AC16 cells (scale bar = 150 µm).

To determine the ability of cCMCMSNs to be taken up by cardiac cells, RhB was loaded onto MSNs, and the so-called “RhB-MSNs” was incubated with AC 16 cells. The results showed that there was no fluorescence signal of RhB in the control group, whereas both RhB-MSNs and RhB-cCMCMSNs showed the red fluorescence signal in the cytoplasmic part (Figure 5a). Notably, cardiac cells treated with RhB-MSNs exhibited a lower red fluorescence signal than that of cells treated with RhB-cCMCMSNs. The percentage of cellular uptake was analysed and showed that the percentage of AC16 cells with a positive RhB signal after RhB-cCMCMSN treatment was 92.00 ± 2.68%, which is significantly higher than the percentage of AC16 cells with the positive RhB signal after RhB-MSN treatment (37.50 ± 14.53%) (Figure 5b). The result showed a significant increase in cellular uptake for cCMCMSNs compared to MSNs. PKH26 was utilised to stain the cCMs to ensure that cCMCMSNs can be coated with cCMs and internalised into cardiac cells. The results indicated that PHK26-cCMCMSNs displayed a red fluorescence signal in the cytoplasmic region (Figure 5c). This is in contrast to the control, which exhibited no signal.

### 3.6. Cell Selectivity of cCMCMSNs on Cardiac Cells

**Figure 6:**
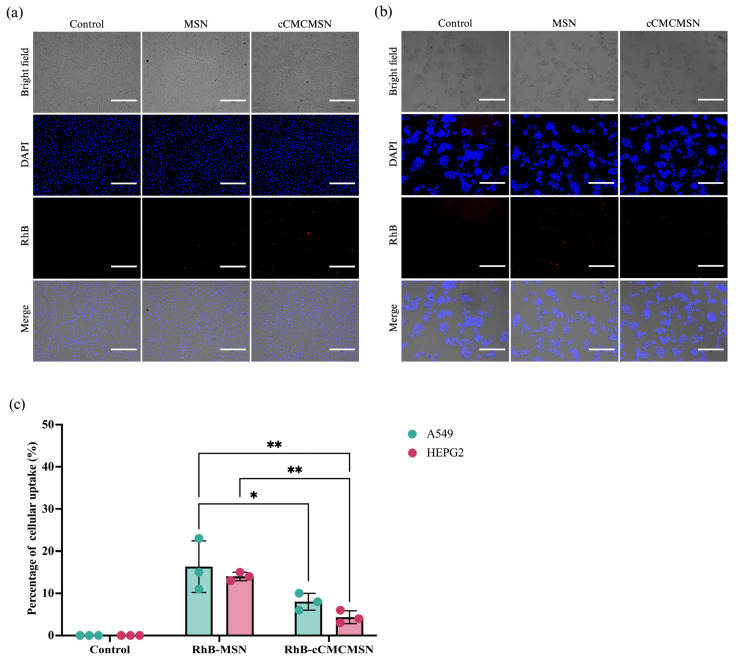
Specificity of cCMCMSNs for A549 and HEPG2: (**a**) internalisation of MSNs and cCMCMSNs by A549 cells, (**b**) internalisation of MSN and cCMCMSNs by HEPG2 cells, (**c**) percentage of cellular uptake by A549 and HEPG2 cells. * *p* < 0.05, ** *p* < 0.01.

The fluorescence imaging in Figure 6 showed the selectivity of cCMCMSNs in lung cells (A549) and liver cells (HEPG2). The results showed that RhB-MSNs can be taken up by two cell types based on the detection of the red fluorescence signal inside the cells. The fluorescence signal was not inside in the lung (Figure 6a) or liver cells (Figure 6b) before treatment and significantly increased after treatment with RhB-cCMCMSNs. Quantitative analysis of the percentage of cellular uptake of RhB-MSNs in A549 and HEPG2 yielded values of 13.00 ± 2.00% and 14.00 ± 1.00%, respectively. In contrast, the uptake of RhB-cCMCMSNs in those 2 cell types yielded values of 8.00 ± 2.00% and 4.33 ± 1.53% (Figure 6), respectively.

### 3.7. Immune Escape of cCMCMSNs

**Figure 7:**
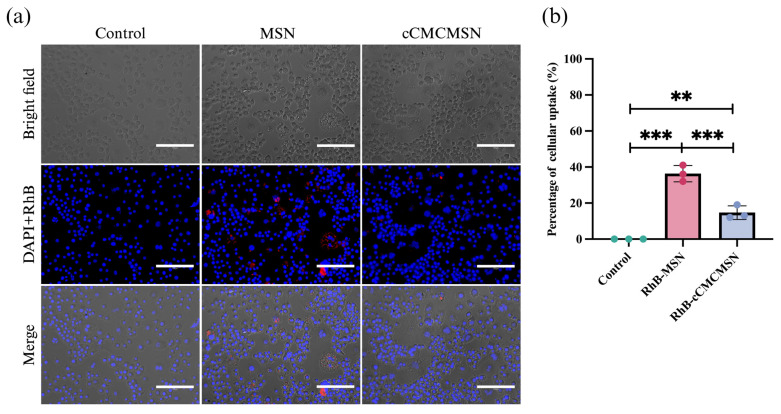
Immune escape of NPs on activated macrophages (RAW 264.7): (**a**) internalisation of NPs by macrophages, (**b**) percentage of internalisation of NPs by macrophage. Scale bar = 150 µm. ** *p* < 0.01, *** *p* < 0.001.

NPs may induce an immune response and activate either the innate or adaptive immune systems in the body. Immune cells, such as macrophages, can recognise these NPs and cause inflammation. However, coating NPs with cell membranes might enhance their ability to mimic natural cells, reducing immune recognition. RAW 264.7 cells were activated to M1 macrophages by treatment with LPS before assessing immune escape. The monocytes were stimulated to activated macrophages after incubation with LPS, as observed from the expression of vacuoles within the cytoplasm (Figure 7a). Both MSNs and cCMCMSNs encapsulating RhB were used in this experiment. The results showed that there was a fluorescence signal of RhB in the MSN-treated group, suggesting phagocytosis of MSNs by macrophages. However, there was less of the fluorescence signal of RhB in macrophages treated with cCMCMSNs. The percentage of NPs phagocytosed by macrophages was 36.33 ± 4.51% for MSNs, which was significantly higher than of 14.67 ± 3.79% for cCMCMSNs (Figure 7b).

## 4. Discussion

The cCMCMSNs were larger than MSNs due to a cCM coating that formed a thin layer around the MSNs. Both cCMs and MSNs had a PDI value range of 0.05–0.7, indicating a moderately uniform size distribution [23]. The PDI value is dependent upon the thickness of the CM coating, leading to a broader size distribution and resulting in an increased PDI. Zeta potential measurements showed that cCMs and MSNs had similar surface charges, with both types of NPs having negative zeta potentials less than −20 mV, which could result in aggregation [24,25]. The nitrogen adsorption–desorption isotherm of MSNs showed a high BET surface area of 959.58 m^2^/g and varying pore sizes, improving the drug adsorption capacity. The membrane protein compositions of MSNs, cCMs, and cCMCMSNs were analysed using SDS-PAGE (Figure 1f). For this analysis, AC16 cells were lysed to obtain whole proteins for comparison with the others. The results from protein electrophoresis showed some of the protein bands were absent from the SDS-PAGE protein separation, which is due to the loss of protein components during the cell membrane purification process. The cardiac cell membrane (cCM) was extracted from human ventricular myocytes cell line (AC16 cells) after cell rupture using hypotonic buffer followed by ultracentrifugation. Therefore, some protein bands that appeared in the cCM lane displayed protein components similar to those present in the whole AC16 cell lysate. However, some protein bands were absent, which is due to the process of purification by ultracentrifugation, and the collected fraction was considered as the membrane fraction. Other cellular components, such as light mitochondria or cytosolic proteins were discarded [8,26]. Notably, MSN displayed no protein bands, indicating the lack of a cell membrane coating. In contrast, cCMCMSNs showed a protein band pattern similar to that of cCMs, indicating that the membrane proteins were mostly retained during the preparation process and that the cell membranes were successfully translocated onto NPs [27]. To confirm the presence of the protein cell membrane marker on cCMCMSNs, Western blotting analysis was conducted on a series of samples, including AC16 cell lysate, MSNs, cCMs, and cCMCMSNs (Figure 1g). A cadherin, a cell membrane marker associated with mediating cell-cell adhesion in animals [28], was employed to analyse the cCM coating. Both cCMs and cCMCMSNs derived from AC16 cells presented cadherin in the Western blot bands compared to the cell lysate, indicating that the cCM was indeed extracted from AC16 cells and that cCMCMSNs were generated by coated NPs with the cCMs.

TEM images revealed the particle shape, showing that MSNs were originally porous spherical particles extracted using CTAB (Figure 2a) [29]. Cardiomyocytes were used to create the vesicles, which displayed a round shape and a double-layered membrane characteristic of the cCM (Figure 2b). The cCMCMSNs presented its spherical shape by incorporating MSN into the cCM structure (Figure 2c). The thick edge might indicate enhanced structural integrity and increased stability, which is essential to preserving the functionality of NPs when they interact with biological systems. In this study, we obtained the full coating of cCMCMSNs and found a partial coating of cCMCMSNs using TEM. The shell integrity of the re-assembled cell membrane coatings can be quantitatively assessed to determine shell integrity using a fluorescence quenching assay, as investigated by Liu et al. They employed fluorescent nitro-2,1,3-benzoxadiazol-4-yl (NBD) labelling of NPs and treated them with dithionite (DT). Their findings revealed that fully coated NPs retained fluorescence after the addition of the DT quencher, indicating intact coatings. Conversely, if the NPs were only partially coated or completely uncoated, the fluorescence intensity diminished gradually due to the reduction of the NBD dye by DT, highlighting the relationship between coating integrity and cellular internalisation potential [27]. However, we did not measure the shell integrity of the re-assembled cell membrane coatings because partially coated NPs could provide the ability for cell internalisation through the presentation of protein and receptor on cell membranes [30].

The encapsulation efficacy of RhB within MSNs and cCMCMSNs was investigated (Figure 3a). These high encapsulation efficiencies suggest that both formulations load the hydrophilic dye RhB extremely effectively, which is crucial for potential applications in imaging (cellular uptake) and drug delivery. RhB can interact with the mesoporous structure of materials particularly through electrostatic interactions. The mesoporous structure provides a large surface area for RhB to interact, which provides high efficiency of RhB loading [31,32]. The high encapsulation efficiencies ensure a significant amount of the drug is released at the target site, which is essential to optimizing therapeutic effects while minimizing toxic effects. RhB was released from MSNs at a faster rate compared to cCMCMSNs, indicating that the coating on the cell membrane might provide a protective barrier to prevent the RhB from being released. A rapid release process through the mesopores that permitted rapid access to the encapsulated RhB might be the cause of the increased initial release from MSNs. RhB diffusion might be impeded by the lipid bilayer or other membrane components, as shown by the delayed release from cCMCMSNs. Therefore, the cCMCMSNs exhibited cumulative drug release properties, which might be beneficial for long-term drug delivery and reduce the frequency of drug administration.

To investigate the applications of cCMCMSNs in biomedicine, we first examined their potential cytotoxicity to cardiac cells (AC16) in vitro. Both MSNs and cCMCMSNs display low cytotoxicity against AC16 cells at the tested concentrations, demonstrating their good biocompatibility, with greater than 90% cell viability. The shell of cCMCMSNs is similar to that of AC16 cells, which can improve biocompatibility, making it a promising candidate for therapeutic applications involving cardiac cells. This suggests that the cell membrane coating on cCMCMSNs might promote biological interactions without inducing adverse reactions, potentially leading to decreased cytotoxicity [33]. However, the preclinical biocompatibility or safety of cCMCMSNs should be evaluated using an in vivo model.

To confirm the penetration of cCMs, PKH26 can be used to label the cCMs on cCMCMSNs, demonstrating that cCMCMSNs can be internalised by interaction with the CM and cardiac cells. The PKH26 dyes are lipophilic, long-chain carbocyanine dyes that are highly fluorescent and are used to stain biological and artificial membranes. The aliphatic tails of these dyes swiftly intercalate into the exposed lipid bilayer, forming strong noncovalent interactions that promote long-term dye retention and stable fluorescence. The results of cellular uptake are shown in Figure 5. When compared to MSN, the data showed a significant increase in cCMCMSN cellular uptake. The significant cellular uptake differential of cardiac cells (above a 55% increase) indicates that the cCMCMSNs are essential for promoting the internalisation of NPs into cardiac cells. This is similar to the report by Xupeng Mu et al., where cell membranes from mesenchymal stem cells (MSCs) were used to coat polydopamine-coated hydrophobic iron oxide NPs (Fe3O4@PDA). Cellular uptake was tested with DU145 cells (prostate cancer cells). The results showed that the Fe3O4@PDA−MSCs NPs had a cellular uptake efficiency of up to 84.2%, which was higher than that of the Fe3O4@PDA NPs, which had an uptake efficiency of 69.2% [34]. The presence of the cell membrane might increase the affinity of the NPs for the target cells, leading to more efficient endocytosis or direct membrane fusion due to the biomimetic properties that the cell membrane coating presents, which makes it easier to interact with cellular receptors. This might be attributed to the existence of lipids or membrane proteins that enhance cellular recognition. The mechanisms of internalisation of cCMCMSNs depend upon receptor-mediated endocytosis, which uses the cell membrane ligands binding to cell surface receptors and requires overcoming the deformation of the cell [35]. The CM possesses characteristics related to extracellular vehicles (EVs); therefore, the internalisation mechanism of cCMCMSNs might be comparable. Receptor-mediated endocytosis is the primary mechanism for binding to receptors on the target cells, as noted in clathrin-dependent endocytosis, lipid raft-mediated endocytosis, micropinocytosis by phagocyte cells, and membrane fusion [36,37,38,39,40]. However, the effect of receptor-mediated endocytosis is relative to the percentage of the cell membrane coating. In contrast, fully coated NPs with more than 50% coating can be internalised individually by the cells, while those with less than 20% coating will be significantly weakened and unable to be internalised by the cells. Lizhi Liu et al. found that internally, partially coated NPs with 20 to 50% coating will aggregate before reaching the cells. Moreover, highly aggregated numbers of NPs were more likely to enter the cell membranes by rotation after entering the membranes to promote more ligand binding with the cell receptor [27]. The advantages for drug delivery, especially cardiovascular therapies, are significant because of the greater uptake of cCMCMSNs. The treatment of cardiovascular diseases relies on the ability to efficiently deliver therapeutic agents to cardiac cells. The results indicated that cCMCMSNs could be a more effective carrier for targeted drug delivery, improving therapeutic efficacy while minimising off-target effects.

The RhB-MSNs and RhB-cCMCMSNs in AC16 cells that showed increased cellular uptake with higher internalisation than A549 and HEPG2, and these agents had significantly higher internalisation than RhB-MSNs. We found that the RhB-cCMCMSNs could selectively internalise into cardiac cells more than the other agents. This result indicated that the selective internalisation of NPs might depend on the expression of homing proteins that are located on the surface of NPs, which are coated with cardiac cell membrane, compared to lung and liver cells. The cardiac cell membrane shell could promote ligand–receptor interactions on cardiac cells and enhance cellular uptake through receptor-mediated endocytosis. The relatively low uptake percentages of RhB-cCMCMSNs in A549 and HEPG2 cells provide support to the idea that cell membrane-coated NPs could be selective for specific cell types. However, cCMCMSNs can still be internalised into lung cells and liver cells, which could be due to the presence of a universal cell membrane protein on the cardiac cell membrane that cross-interacts with lung and liver cells. Moreover, the lipid bilayer structure of the cell membrane could also provide the fusion between cCMCMSNs and other cell types. This explanation is related to the study by Lizhi Liu et al. They investigated the specificity of colon carcinoma cell (CT2) cell membrane-coated SiO_2_ NPs (CM-SiO_2_ NPs) in different three types of cells, including CT26, cervical carcinoma (HeLa), and breast cancer (MCF-7) cells. They suggested that CM-SiO_2_ NPs exhibited 1.6- to 2-fold greater internalisation by CT26 cells than Hela and MCF-7 cells [27]. However, internalisation in different cell types might depend on the dose of NPs [41]. Thus, the significantly greater uptake of cCMCMSNs by cardiac cells indicated the potential of this compound for targeted drug delivery in cardiac applications. Due to its reduced off-target effects, this selectivity or specificity might improve the therapeutic efficacy of heart treatments.

The recognition of NPs by immune cells, particularly macrophages, can trigger an inflammatory response. The response could decrease the effectiveness of drugs, affecting distribution, metabolism, and elimination. Immune escape strategies might improve therapeutic efficacy and diminish adverse effects by augmenting the effective dosage of drugs delivered to the target region. In this study, the immune escape of MSNs and cCMCMSNs was evaluated using RAW 264.7 cells, which were activated into M1 macrophages. The result showed activated M1 macrophages could phagocytose MSNs more than cCMCMSNs obviously, indicating that immune cells might recognise and phagocytose uncoated NPs more easily. MSNs cannot be completely phagocytosed because of their nanoscale size and unique surface properties. NPs can avoid excessive clearance by the immune system [42]. cCMCMSNs showed significantly reduced phagocytosis, indicating that the cell membrane coating might provide protection against recognition and internalisation by activated macrophages. The similarity of cCMCMSNs to native cell membranes might reduce macrophage activation or change the expression of surface receptors involved in the uptake of NPs, resulting in a reduction in phagocytosis [27]. However, M1 macrophages could still phagocytose cCMCMSNs because partially coated NPs can include an uncoated region that immune cells can recognise and phagocytose.

This study showed the fabrication of cardiac cell membrane-coated mesoporous silica nanoparticles (cCMCMSNs) for selective delivery to cardiac cells with an enhanced ability to avoid phagocytosis by immune cells. The major findings of the current study show that cCMCMSNs can control drug release, reduce cytotoxicity, enhance selective uptake by cardiac cells, and reduce macrophage phagocytosis. These findings highlight the promising potential of cCMCMSNs in the field of targeted drug delivery, particularly in cardiac therapy.

A significant issue arises that pertains to a limitation of the study, as it relied exclusively on an in vitro model. This study presents an in vitro proof-of-concept for utilising cardiac cell membranes to modify the surface of nanoparticles, thereby improving selective delivery to target cells or tissues, which may not have further physiological implications. However, it is essential to assess the efficiency of targeting in an in vivo model utilising a small animal imaging system to track the labelled-cCMCMSNs. Furthermore, to evaluate the safety and clinical impact of cCMCMSN-mediated drug delivery, it is necessary to load therapeutic agents into the cCMCMSNs and assess their cardioprotective effects on cardiac cells under pathological conditions, such as ischaemia/reperfusion injury, post-ischaemic cardiac remodelling and hypertrophy, inflammatory cardiomyopathy, and cardiotoxicity from chemotherapy. This could provide not only functional information related to disease in humans but also some mechanistic insight into targeted therapy in cardiovascular diseases. More importantly, the long-term effect of targeting delivery using cCMCMSNs, both in small and large animal models, should also be determined to provide safety information that is crucial for further investigations in clinical trials.

## 5. Conclusions

In conclusion, this study demonstrated the advancement and enhancement of cardiac selective delivery of nanoparticles by utilising the cardiac cell membrane to coat mesoporous silica nanoparticles (cCMCMSNs). The cCMCMSNs presented excellent characteristics as drug carriers, including high encapsulation efficiency and controlled release capability. For the treatment of cardiac cells, the cCMCMSNs exhibited low toxicities and improved its targeted delivery to these cells. Furthermore, the cCMCMSNs could reduce phagocytosis by immune cells, which constitutes the main inflammatory response. These properties could indicate the advantages of nanoparticles for targeted cardiac delivery and offer an alternative approach for surface modification for targeted drug delivery.

## Data Availability

The data that support the findings of this study are available from the corresponding author, Associate Professor Dr. Sarawut Kumphune, upon reasonable request.

## Abbreviations

NPs	Nanoparticles
cCM	Cardiac cell membrane
MSNs	Mesoporous silica nanoparticles
cCMCMSNs	Cardiac cell membrane-coated mesoporous silica nanoparticles
